# False Opposing Fear Memories Are Produced as a Function of the Hippocampal Sector Where Glucocorticoid Receptors Are Activated

**DOI:** 10.3389/fnbeh.2020.00144

**Published:** 2020-08-26

**Authors:** Nadia Kaouane, Eva-Gunnel Ducourneau, Aline Marighetto, Menahem Segal, Aline Desmedt

**Affiliations:** ^1^INSERM, Neurocentre Magendie, Physiopathologie de la Plasticité Neuronale, Bordeaux, France; ^2^University of Bordeaux, Bordeaux, France; ^3^Department of Neurobiology, The Weizmann Institute, Rehovot, Israel

**Keywords:** dorsal hippocampus, ventral hippocampus, fear memory, fear conditioning, glucocorticoid receptors, mice

## Abstract

Injection of corticosterone (CORT) in the dorsal hippocampus (DH) can mimic post-traumatic stress disorder (PTSD)—related memory in mice: both maladaptive hypermnesia for a salient but irrelevant simple cue and amnesia for the traumatic context. However, accumulated evidence indicates a functional dissociation within the hippocampus such that contextual learning is primarily associated with the DH whereas emotional processes are more linked to the ventral hippocampus (VH). This suggests that CORT might have different effects on fear memories as a function of the hippocampal sector preferentially targeted and the type of fear learning (contextual vs. cued) considered. We tested this hypothesis in mice using CORT infusion into the DH or VH after fear conditioning, during which a tone was either paired (predicting-tone) or unpaired (predicting-context) with the shock. We first replicate our previous results showing that intra-DH CORT infusion impairs contextual fear conditioning while inducing fear responses to the not predictive tone. Second, we show that, in contrast, intra-VH CORT infusion has opposite effects on fear memories: in the predicting-tone situation, it blocks tone fear conditioning while enhancing the fear responses to the context. In both situations, a false fear memory is formed based on an erroneous selection of the predictor of the threat. Third, these opposite effects of CORT on fear memory are both mediated by glucocorticoid receptor (GR) activation, and reproduced by post-conditioning stress or systemic CORT injection. These findings demonstrate that false opposing fear memories can be produced depending on the hippocampal sector in which the GRs are activated.

## Introduction

Exposure to an extreme stress can produce highly fearful memories, which contribute to the development of stress-related disorders (de Quervain et al., 1998). In such a situation, excess glucocorticoids, whose the hippocampus constitutes a key brain site of action, impair hippocampus-dependent memory consolidation of the event (McEwen, 2000; Roozendaal, 2003). Therefore, this may explain the emergence of pathological fear memories like those observed in post-traumatic stress disorder (PTSD; Layton and Krikorian, 2002; Desmedt et al., 2015a). In accordance with this, we previously demonstrated in mice that, under a high stressful situation, post-training infusion of glucocorticoids into the dorsal hippocampus (DH) impairs contextual fear memories while inducing fear memory for an irrelevant (i.e., not predicting the threat) simple tone, thereby mimicking both contextual amnesia and the maladaptive hypermnesia observed in PTSD (Kaouane et al., 2012).

However, accumulated evidence demonstrates a functional dissociation along the dorsal–ventral axis of the hippocampus (Fanselow and Dong, 2010). The DH receives polymodal sensory information from cortical areas (Witter and Amaral, 2004) and primarily contributes to contextual learning. In contrast, the ventral hippocampus (VH) is strongly connected to the subcortical structures, especially the amygdala, and may rather contribute to emotion-related processes (Moser and Moser, 1998; Bannerman et al., 2004), particularly fear conditioning (Maren, 1999; Bast et al., 2001; Maren and Holt, 2004) and anxiety-related behaviors (Bannerman et al., 2004; Calhoon and Tye, 2015). In addition, stress and glucocorticoids differentially regulate long-term potentiation (LTP) and long-term depression (LTD) in the DH and the VH (Maggio and Segal, 2007a, b, 2009a,b). In particular, they impair LTP in the DH while enhancing it in the VH (Maggio and Segal, 2007b), whereas stress increases LTD in the DH while converting LTD to LTP in the VH (Maggio and Segal, 2009a).

Moreover, the glucocorticoid receptors (GRs), on which depend the glucocorticoids’ memory effects (Oitzl et al., 2001), show a higher density in the DH than in the VH (Robertson et al., 2005; Segal et al., 2010). Of particular interest in the context of normal vs. pathological fear memory, GR activation within the hippocampus is crucial for the consolidation of contextual fear memories and can even enhance them (Donley et al., 2005; Revest et al., 2005, 2010, 2014) whereas full GR activation results in impaired spatial memory (Conrad et al., 1999; Brinks et al., 2007). Furthermore, specific activation of GRs also abolishes *in vitro* synaptic excitability in both DH and VH (Segal et al., 2010) and facilitates LTD in both hippocampal sectors (Maggio and Segal, 2009a). Together, these data strongly suggest that GR activation in either DH or VH could differentially contribute to the deleterious effects of glucocorticoids on fear memories.

To address this issue: (1) we compared the effects of local infusions of corticosterone (CORT), the major glucocorticoid in rodents, into either the DH or the VH, on the consolidation of tone and contextual fear memories; (2) we assessed whether these effects are mediated by GR activation; and (3) we tested whether these effects could be physiologically mimicked by post-training stress or systemic CORT injection.

## Materials and Methods

### Subjects

Three-month-old male mice (C57Bl/6 JI Company, Charles River Laboratories) were individually housed a week before experiments in standard Macrolon cages in a temperature- and humidity-controlled room under a 12-h light/dark cycle (lights on at 07:00) and had *ad libitum* access to food and water. As all the present experiments were restricted to male mice, future experiments will have to determine the extent to which the present findings can be extended to female mice. Mice were handled a few days before experiments and habituated to intracerebral or systemic injection procedures. All experiments took place during the light phase. All animal care and behavioral tests were conducted in compliance with the European Communities Council Directive (86/609/EEC).

### Surgical Procedure

Mice were anesthetized with ketamine (80 mg/kg body weight, i.p.) and xylazine (16 mg/kg body weight, i.p.; Bayer) and secured in a David Kopf Instruments stereotaxic apparatus. Stainless-steel guide cannulas (26 gauge, 8-mm length) were implanted bilaterally 1 mm above either the dorsal hippocampus (A/P, −2 mm; M/L, ±1.3 mm; D/V, 1 mm) or the ventral hippocampus (A/P, −3.6 mm; M/L, ±3 mm; D/V, 3.3 mm; relative to dura and bregma; Franklin and Paxinos, 1997), then fixed in place with dental cement and two jeweler screws attached to the skull. Mice were then allowed to recover in their home cage for at least 8 days before behavioral experiments.

### Fear Conditioning Procedures

**Figure 1:**
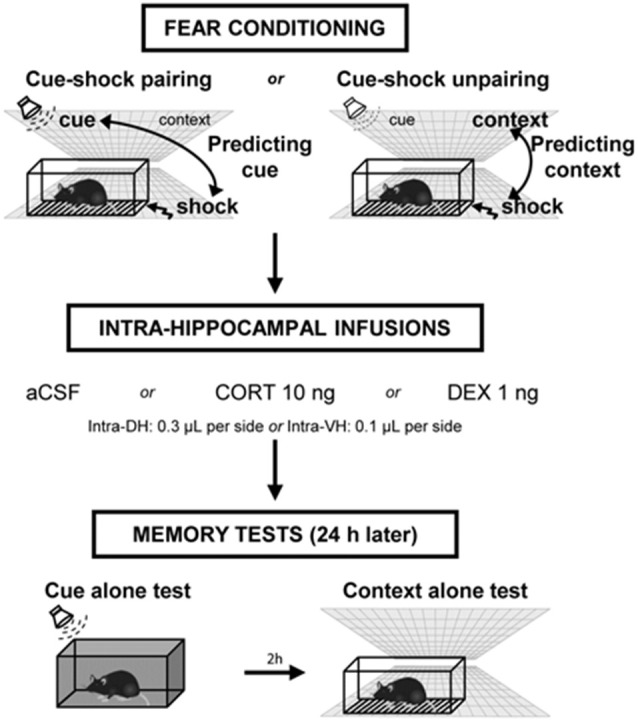
Experimental design of the behavioral procedure. Mice were either submitted to a cue–shock *pairing* procedure (predicting-cue group) or to a cue–shock *unpairing* procedure (predicting-context group). Immediately after conditioning, animals received intra-hippocampal infusions of artificial cerebrospinal fluid (aCSF), corticosterone (CORT), or dexamethasone (DEX). The next day, mice were first re-exposed to the cue alone in a neutral chamber, then (2 h later) were re-exposed to the conditioning context without the cue. During these tests, the freezing responses were measured during 2-min periods.

The procedures have been fully described in previous studies (Calandreau et al., 2005, 2006, 2007; Kaouane et al., 2012). Briefly, mice were placed in the conditioning chamber and, after a baseline period of 100 s, received two tone cues (63 dB, 1 kHz, 15 s) either paired (intertrial interval of 60 s) or unpaired (pseudo-random distribution of the stimuli) with two electric footshocks (0.8 mA, 50 Hz, 3 s). With the tone presentation always followed by the shock delivery (cue–shock *pairing* procedure), the animals identified the tone as the main threat predictor of the shock (predicting-cue group). In contrast, when the tone presentation was never followed by shock delivery (cue–shock *unpairing* procedure), the animals identified the conditioning context as the right predictor of the shock (predicting-context group; Figure 1). Specifically, in the CS–US unpairing procedure, 100 s after being placed into the chamber, animals received a shock, then, after a 20-s delay, a tone; finally, after a 30-s delay, the same tone and the same shock spaced by a 30-s interval were presented. After 20 s, animals were returned to the home cage. The relatively high footshock intensity used (i.e., 0.8 mA) is known to produce a strong fear conditioning to the tone or to the context in the predicting-cue group and the predicting-context group, respectively, as demonstrated in our previous study (Kaouane et al., 2012). The quality of the memory formed was assessed the following day when mice were submitted to two memory tests. First, after a 2-min baseline period in a neutral chamber, mice were exposed for 2 min to the tone cue alone, followed by a 2-min post-tone period. Conditioned fear to the tone is expressed by the percentage of freezing during the tone presentation, and the strength and specificity of this conditioned fear is attested by a ratio that considers the percentage of freezing increase to the tone with respect to a baseline freezing level (i.e., pre- and post-tone periods mean) and that was calculated as follows: [% freezing during tone presentation − (% pre-tone period freezing + % post-tone period freezing)/2]/[% freezing during tone presentation + (% pre-tone period freezing + % post-tone period freezing)/2]. Two hours later, mice were re-exposed for 6 min to the conditioning context alone for the assessment of their contextual freezing, which attests of their contextual memory of the aversive event. As for the tone test, the percentage of freezing is assessed during three 2-min blocks. However, because all contextual fear responses decline on the second and third 2-min blocks of the context test because of a classical fear extinction and that the first 2-min block is the only one that allows the observation of key optimal differences between the different groups, we chose to restrict the data to the first block. Freezing behavior of animals, defined as a lack of all movement except for respiratory-related movements, was used as an index of conditioned fear response. Animals were continuously recorded on videotape for off-line second-by-second scoring of freezing by an observer blind of experimental groups.

### Intracerebral Infusions

Immediately after the acquisition of fear conditioning, animals were placed back in their home cage and received intra-DH or intra-VH bilateral infusions (0.3 or 0.1 μl per side, respectively) of artificial cerebrospinal fluid (aCSF), CORT (2-hydroxypropyl-β-cyclodextrin complex; Sigma–Aldrich; 10 ng per side), or the specific GR agonist dexamethasone (DEX: 2-hydroxypropyl-β-cyclodextrin complex; Sigma–Aldrich; 1 ng per side). The dose of CORT was selected based on our previous study reporting that post-training intra-DH infusions of 10 ng disturbed the selection of the right predictor of the shock under a 0.8-mA footshock intensity (Kaouane et al., 2012). The dose of DEX was selected on the basis of previous *in vivo* studies using similar concentration for intra-DH infusions in rats (Mizoguchi et al., 2007) and reporting that dose 10-fold lower than for CORT is efficient to mimic *in vitro* CORT effects (Maggio and Segal, 2007b; Chaouloff et al., 2008). For infusions, stainless-steel cannulas (32-gauge, 9 mm) attached to 1-μl Hamilton syringes with polyethylene catheter tubing were inserted through the guide cannula. The syringes were fixed in a constant rate infusion pump (0.1 μl/min). The cannulas were left in place for an additional 1 min before removal to guarantee diffusion of the drug.

### Restraint Stress

Immediately after the acquisition of fear conditioning using a footshock of 0.8 mA or a lower one (0.3 mA) known to be too weak for inducing significant fear responses but with which post-training stress can enhance contextual fear conditioning (Kaouane et al., 2012), mice were placed during 20 min in a transparent Plexiglas cylinder (diameter: 2.5 cm, 11 cm long) in a room adjacent to the fear conditioning room. After immobilization, mice were returned to their home cage.

### Systemic Injection of Corticosterone

CORT (2-hydroxypropyl-β-cyclodextrin complex) or vehicle (NaCl 0.9%) was administrated intraperitoneally (i.p.) immediately after the acquisition of fear conditioning. The complex of corticosterone with cyclodextrins allows dissolving this steroid in aqueous solutions. After the injection, animals were returned to their home cage. We selected a relatively low (1.5 mg/kg) and a high (10 mg/kg) dose of corticosterone (in a volume of 0.1 ml/10 g bodyweight) known to produce dose-dependent effects on fear memories as shown in our previous study (Kaouane et al., 2012).

### Histology

After behavioral testing, animals were given an overdose of pentobarbital and transcardially perfused with physiological saline, followed by 10% buffered formalin. Brains were postfixed in formalin–sucrose 30% solution for 1 week, frozen, cut coronally on a sliding microtome into 60-μm sections that were mounted on gelatin-coated glass slides, and stained with thionine to evaluate the cannula placements (Supplementary Figure S1).

### Data Analysis

Data are presented as the mean ± SEM. Statistical analyses were performed using, on StatView software, analysis of variance (ANOVA) followed by Bonferroni–Dunn *post hoc* test when appropriate. Values of *p* < 0.05 were considered as significant. Several experiments using a similar behavioral protocol led to the conclusion that 6–8 mice per group were enough to reach statistical significance. To face the variability of surgery, we increased the sample size to 10 mice per group. For indication, the power of these experiments, with *n* = 8 mice per group and an alpha of 0.05, is 85%.

## Results

### Activation of GR in the DH Produces a Simple Tone-Based Fear Memory at the Expense of Contextual Fear Memory

**Figure 2:**
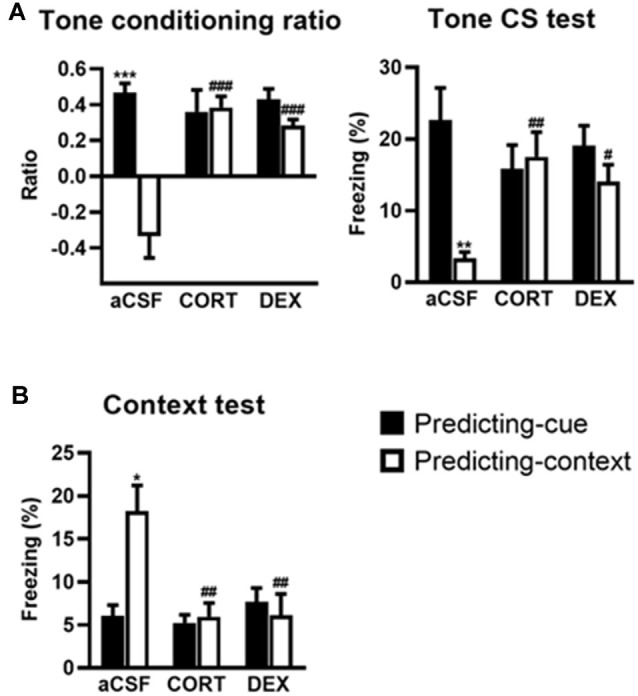
Activation of glucocorticoid receptor (GR) in the dorsal hippocampus (DH) produces a simple tone-based fear memory at the expense of contextual fear memory. In controls (aCSF), mice of the predicting-cue group (*n* = 7) expressed a high tone conditioning ratio (**A**, *left*) and high percentage of freezing to the tone *per se* (**A**, *right*), as well as low freezing to the context **(B)**, in comparison with the predicting-context group (*n* = 8), which expressed low levels of freezing to the cue and high levels of freezing to the context. In mice submitted to the predicting-context condition, intra-DH CORT or DEX infusions increased the tone conditioning ratio (**A**, *left*), produced a fear response to the cue (**A**, *right*), while reducing the conditioned fear to the context (**B**; CORT: *n* = 7; DEX: *n* = 6). No change was observed in mice submitted to the predicting-cue condition (CORT: *n* = 7; DEX: *n* = 9). In all conditions, the ratio differed from zero (all *p* < 0.05). *Procedure effect (predicting cue vs. predicting context group; *p < 0.05, **p < 0.01, and ***p < 0.001); ^#^treatment effect (aCSF vs. CORT or aCSF vs. DEX; ^#^*p* < 0.05, ^##^*p* < 0.01, and ^###^*p* < 0.001).

This experiment first replicates our previous findings showing that intra-DH injection of CORT mimicked a simple/salient tone fear conditioning at the expense of contextual conditioning, thereby reproducing the PTSD-like pathological hypermnesia and contextual amnesia (Kaouane et al., 2012). Second, the present results reveal that such abnormal fear memory can also be produced by intra-DH injection of DEX, indicating that GR activation is sufficient to induce such memory alteration. The effects of the intra-DH injections of CORT and DEX on the fear responses to the tone (Figure 2A) and the conditioning context (Figure 2B) were dependent on the conditioning procedure (i.e., predicting-cue vs. predicting-context) as they were both restricted to the predicting-context condition [Tone conditioning ratio: effect of treatment (*F*_(2,38)_ = 8.19, *p* < 0.01) and treatment × procedure (*F*_(2,38)_ = 13.00, *p* < 0.001); Context test: effect of treatment (*F*_(2,38)_ = 6.20, *p* < 0.01) and treatment × procedure (*F*_(2,38)_ = 7.04, *p* < 0.01)].

First, compared with their aCSF-injected controls, CORT- and DEX-injected mice submitted to the predicting-context procedure displayed increased fear responses to the tone, attested by a significant increased tone conditioning ratio (Figure 2A, left, both CORT and DEX: Bonferroni–Dunn *post hoc* test *p* < 0.001), and a significant increase in the percentage of freezing level to the tone *per se* (Figure 2A, right, CORT: *p* < 0.01, DEX: *p* < 0.05; see also Supplementary Figure S2A), so that they did not differ anymore from animals submitted to the predicting-tone procedure (in CORT and DEX groups: both *p* > 0.05). It must be noted that in our previous study (Kaouane et al., 2012), we also showed that CORT-injected mice displayed a fear response to a previously unexperienced cue (2-kHz tone) at some extent similar to the one experienced during the conditioning (1-kHz tone), but not to a completely different cue (white noise). Thus, as in PTSD, these mice showed a partial fear generalization to cues more or less similar to trauma-related cues, but not to very different cues.

In parallel, although the conditioning context is the objective predictive stimulus in this training condition, the same mice displayed significantly decreased contextual fear responses compared with aCSF controls (Figure 2B, both CORT and DEX: Bonferroni–Dunn *post hoc* test *p* < 0.01). As a result, their levels of contextual freezing were as low as those displayed by mice submitted to the predicting-tone condition (in CORT and DEX groups: *p* > 0.05).

### Activation of GR in the VH Promotes a Context-Based Fear Memory at the Expense of Tone-Based Fear Memory

**Figure 3:**
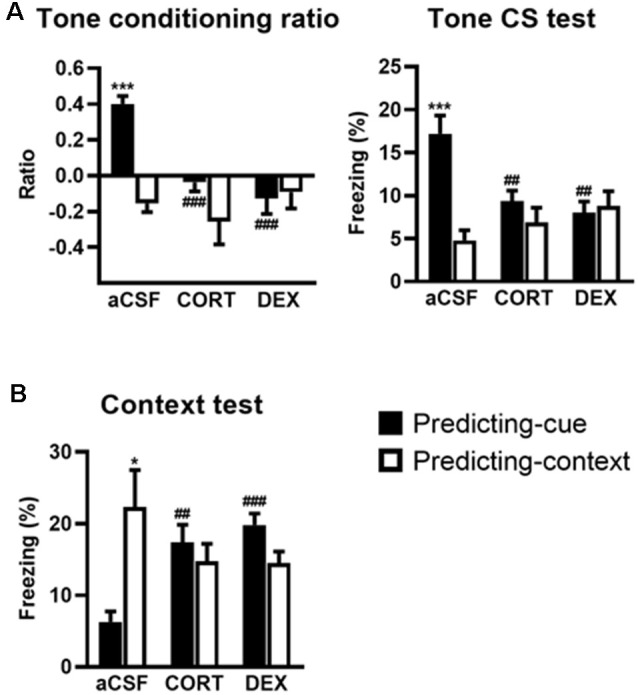
Activation of GR in the ventral hippocampus (VH) promotes a context-based fear memory at the expense of tone-based fear memory. The controls (aCSF) expressed a high tone conditioning ratio (**A**, *left*) with high percentage of freezing to the tone *per se* (**A**, *right*; predicting-cue group: *n* = 8) and low conditioned fear to the context **(B)** when submitted to the predicting-cue condition (*n* = 8), whereas they expressed an inverse pattern of results when submitted to the predicting-context condition (*n* = 8). In the predicting-cue condition, intra-VH CORT or DEX infusions (CORT: *n* = 8; DEX: *n* = 8) abolished the tone conditioning ratio (**A**, *left*) and the conditioned fear to the tone *per se* (**A**, *right*), while increasing the fear responses to the context **(B)**. After CORT and DEX infusions, the ratios did not differ from zero (all *p* > 0.05). No change was observed in mice submitted to the predicting-context condition (CORT: *n* = 7; DEX: *n* = 7). *Procedure effect (predicting cue vs. predicting context group; *p < 0.05 and ***p < 0.001); ^##^treatment effect (aCSF vs. CORT or aCSF vs. DEX; ^##^*p* < 0.01 and ^###^*p* < 0.001).

The effects of intra-VH injections of CORT and DEX on the fear responses to the tone (Figure 3A) and the conditioning context (Figure 3B) were dependent on the conditioning procedure (i.e., predicting-cue vs. predicting-context), but in contrast to intra-DH injections, they were both restricted to the predicting-cue condition [Tone conditioning ratio: effect of treatment (*F*_(2,40)_ = 7.26, *p* < 0.01) and treatment × procedure (*F*_(2,40)_ = 7.40, *p* < 0.01); Context test: effect of treatment × procedure (*F*_(2,40)_ = 8.61, *p* < 0.001)].

First, compared with their aCSF-injected controls, CORT- and DEX-injected mice submitted to the predicting-cue procedure did not express any conditioned fear to the tone, which is the objective predictive stimulus in this training condition. This blockade is attested by a significant decreased tone conditioning ratio (Figure 3A, left, both CORT and DEX: Bonferroni–Dunn *post hoc* test *p* < 0.001) and a significant decrease in the percentage of freezing level to the tone *per se* (Figure 3A, right, both CORT and DEX: *p* < 0.01; see also Supplementary Figure S2B). As a result, these mice did not differ anymore from those submitted to the predicting-context procedure (in CORT and DEX groups: both *p* > 0.05).

In parallel, the same mice displayed significantly increased fear responses to the conditioning context (Figure 3B, CORT: *p* < 0.01, DEX: *p* < 0.001) to the extent that their levels of contextual freezing were as high as those of mice submitted to the predicting-context condition (in CORT and DEX groups: both *p* > 0.05).

### Stress or Systemic CORT Injection Mimics the Effects of Local CORT Infusion on Fear Memories

**Figure 4:**
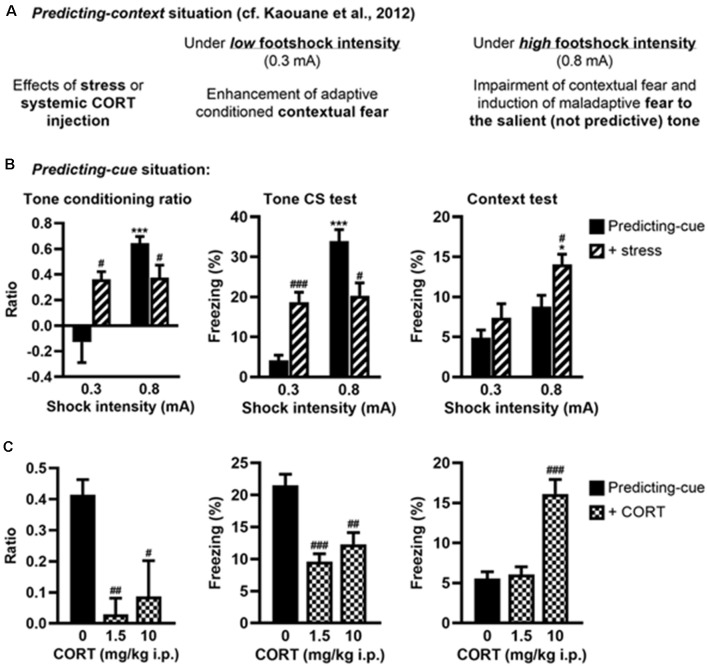
Stress or systemic CORT injection mimics the effects of local CORT infusion on fear memories. **(A)** Summary of previously published data relative to the effects of post-training stress or systemic CORT injection on fear memory after a *predicting-context* procedure. **(B)** Post-training stress after a *predicting-cue* procedure using a low shock intensity (0.3 mA) increased the tone conditioning ratio (*left*) and the fear responses to the tone *per se* (*middle*), whereas the same stress applied after this conditioning procedure using a high shock intensity (0.8 mA) reduced them and significantly increased the fear responses to the context (*right*). 0.3 mA control: *n* = 8; 0.3 mA + stress: *n* = 9; 0.8 mA control: *n* = 9; 0.8 mA + stress: *n* = 8. **(C)** Intraperitoneal (i.p.) corticosterone injection after a 0.8-mA predicting-cue conditioning decreased the tone conditioning ratio (*left*), the fear responses to the tone *per se* (*middle*), and increased at 10 mg/kg the fear responses to the context (*right*). After CORT injection, the ratios decreased such that it did not differ from zero (both *p* > 0.05). Control (0): *n* = 9; CORT 1.5: *n* = 10; CORT 10: *n* = 8. *Effect of shock intensity (0.3 vs. 0.8 mA; *p < 0.05 and ***p < 0.001); ^#^effect of stress or injection compared with controls (^#^*p* < 0.05, ^##^*p* < 0.01, and ^###^*p* < 0.001).

We previously showed that post-training (restraint) stress or systemic CORT injection performed after a *predicting-context* conditioning mimicked the effects of intra-DH CORT infusions on fear memories, i.e., promoting the selection of the tone cue instead of the context as predictor of the shock when a relatively high stress intensity was used (Kaouane et al., 2012, and see Figure 4A for a summary). Here, in the same perspective of more physiological stress-related manipulations, we tested whether similar manipulations performed after a *predicting-cue* conditioning could mimic the effects of intra-VH CORT infusions on fear memories, i.e., the selection of the context instead of the tone cue as predictor of the shock.

First, we analyzed whether post-training stress had differential effects on tone fear conditioning depending on the shock intensity, as demonstrated for contextual fear conditioning (Kaouane et al., 2012). The amplitude of the conditioned fear to the tone (Figure 4B, left and middle, Supplementary Figure S2C) was dependent on the footshock intensity (0.3 vs. 0.8 mA; effect of intensity on tone conditioning ratio: *F*_(1,30)_ = 16.26, *p* < 0.001; on freezing to the tone *per se*: *F*_(1,30)_ = 37.47, *p* < 0.001), and depending on this intensity the post-training stress had an opposite effect on the tone conditioning (intensity × stress for tone conditioning ratio: *F*_(1,30)_ = 15.21, *p* < 0.001; for freezing to the tone *per se*: *F*_(1,30)_ = 30.17, *p* < 0.001). As expected, the conditioned fear responses to the tone were higher after a footshock of 0.8 mA than 0.3 mA in the control condition (Bonferroni–Dunn *post hoc* test *p* < 0.001), but this difference disappeared in the post-training stress condition because both the tone conditioning ratio and the percentage of freezing to the tone increased after a 0.3-mA footshock (*p* < 0.05 and *p* < 0.001, respectively) whereas they decreased after a 0.8-mA footshock (both *p* < 0.05). In parallel, the amplitude of the fear responses to the conditioning context (Figure 4B, right) increased with the footshock intensity (effect of intensity: *F*_(1,30)_ = 14.12, *p* < 0.001) and the post-training stress (effect of stress: *F*_(1,30)_ = 7.7, *p* < 0.01), which produced a significant enhancement of contextual freezing when the highest footshock intensity was used during training (*p* < 0.05).

Second, we analyzed whether systemic (i.p.) injection of CORT could mimic the deleterious effects of intra-VH CORT injections on fear memory when performed after tone fear conditioning using a 0.8-mA footshock (Figure 4C). Systemic CORT injection decreased both the tone conditioning ratio (Figure 4C, left, treatment: *F*_(2,24)_ = 8.13, *p* < 0.01) and the percentage of freezing to the tone *per se* (Figure 4C, middle, treatment: *F*_(2,24)_ = 15.82, *p* < 0.001; see also Supplementary Figure S2D), whatever the dose used (1.5 mg/kg: *p* < 0.01 and *p* < 0.001, respectively; 10 mg/kg: *p* < 0.05 and *p* < 0.01, respectively), but increased the fear responses to the conditioning context (Figure 4C, right, treatment: *F*_(2,24)_ = 23.11, *p* < 0.001) at 10 mg/kg (*p* < 0.001). Therefore, peripheral CORT administrations after tone fear conditioning dose-dependently reproduced the effects of local VH injections.

## Discussion

The present results show that CORT differentially alters fear memories depending on the hippocampal sector where GRs are activated and the fear learning considered. Replicating our previous results (Kaouane et al., 2012), intra-DH CORT infusion impaired contextual fear conditioning and induced fear responses to a salient cue, non-predicting the threat. Strikingly, the present study shows that intra-VH infusion produced the exact opposite pattern: it blocked cue fear conditioning while inducing fear responses to the (background) conditioning context which was not yet the main predictor of the shock. The fact that these opposite effects could be reproduced by local infusions of DEX indicates that they are mediated by activation of GRs in the DH and VH, respectively. Finally, post-training stress or systemic CORT injections reproduced the alterations of fear memories induced by local infusions of glucocorticoids.

The replication of the deleterious effect of intra-DH infusion of CORT on contextual fear memory is fully congruent with a vast literature indicating that the dorsal part of the hippocampus supports the establishment of a unified representation of the context (Rudy et al., 2002; Matus-Amat et al., 2004) and is crucial for contextual fear conditioning (Kim and Fanselow, 1992; Phillips and LeDoux, 1992; Anagnostaras et al., 2001). In accordance with previous studies, our past and present results also support the idea that CORT can have different effects on hippocampus-dependent memories, promoting contextual fear memories under low stress situations (Pugh et al., 1997; Cordero and Sandi, 1998; Revest et al., 2005, 2010, 2014; Kaouane et al., 2012), while disrupting spatial (de Quervain et al., 1998; Conrad et al., 1999) and contextual memory (Kaouane et al., 2012) when high doses or high stress situations are used. Interestingly, intra-DH infusion of CORT also resulted in the selection of the simple tone instead of contextual cues as predictor of the shock, leading to a prevalent, although maladaptive, tone-based fear memory. Similar switch from contextual to cue fear conditioning was already observed after pharmacological manipulations that reduced the dorsal hippocampal activity (Calandreau et al., 2006; Desmedt et al., 2015b). This indicates that, when the consolidation of predicting contextual information is disrupted by alteration of the DH, a cognitive switch promotes an association between the footshock and the most salient simple cue (i.e., the tone), despite the absence of any explicit cue–shock pairing during training.

In contrast, when CORT was infused into the VH, the present study shows that tone cue conditioning was blocked to the benefit of contextual fear. Conditioned fear to a discrete tone is classically viewed to involve a brain circuit restricted to the amygdala and the thalamus (LeDoux, 2000). However, numerous studies have reported that tone cue conditioning can be impaired by electrolytic (Maren and Holt, 2004), neurotoxic lesions (Maren, 1999; Bast et al., 2001; Zhang et al., 2001), or inactivation of the VH (Maren and Holt, 2004; Esclassan et al., 2009). Because the VH is strongly connected to the amygdala (Maren and Fanselow, 1995; Pitkänen et al., 2000), it could convey information about the tone to it (Sakurai, 2002). This transmission would be here disrupted by excess glucocorticoids in the VH.

In parallel, intra-VH CORT infusion also increased conditioned fear to the context in animals for which the tone is yet the objective predictor of the shock, and as such known to normally overshadow contextual cues. The role of the VH in contextual fear conditioning is unclear because it receives little visuo-spatial information from the sensory cortices (Pitkänen et al., 2000; Witter and Amaral, 2004), displays less numerous and less specific place cells than the DH (Jung et al., 1994), and its lesion or inactivation resulted in opposite results (Bast et al., 2001; Zhang et al., 2001; Kjelstrup et al., 2002; Hunsaker and Kesner, 2008). It could thus be hypothesized that CORT-induced alteration of the VH could promote cognitive processes based on the DH functioning. Particularly, previous data have shown that promoting the activity of the DH can abolish tone fear conditioning while promoting background contextual conditioning (Calandreau et al., 2006), mimicking the present effects of intra-VH CORT infusions. Therefore, our results suggest that when a simple cue–shock association is blocked by interfering with VH-dependent processes, a context–shock association, which could be mainly supported by the DH, is preferentially consolidated, leading to a prevalent contextual fear memory even if the context, which is consigned in the background in this learning situation, is not the best predictor of the threat.

The present study also shows that activation of the same receptor (GRs), by infusion of the specific agonist DEX either into the DH or the VH, mimicked the opposite CORT-induced alterations of fear memories. CORT can act on two subtypes of nuclear receptors: the high-affinity mineralocorticoid receptor (MR) and the low-affinity GR (de Kloet et al., 1998). Both receptors are found in the hippocampus and often co-localized on the same neurons (Joëls, 2007). At the basal level, only MRs are occupied. During mild stress, the increase in CORT levels results in full MR and moderate GR occupancy, resulting in enhanced synaptic plasticity in the hippocampus (Diamond et al., 1992). This effect is thought to mediate the facilitating effects of glucocorticoids on hippocampus-dependent memory function (Conrad et al., 1999; Brinks et al., 2007). In contrast, full GR occupancy, which occurs during high stressful situation, impairs synaptic excitability and hippocampus-dependent memory functions (Conrad et al., 1999; Brinks et al., 2007). Specifically, GR activation in the DH was shown to disrupt excitability and synaptic plasticity (Diamond et al., 1992; Kim and Yoon, 1998; Garcia, 2001; Maggio and Segal, 2007b), which is crucial for contextual fear conditioning (Sacchetti et al., 2001). In the VH, whereas stress or direct corticosterone bath application promotes LTP *via* the MRs, specific activation of GRs results in low excitability and synaptic plasticity (Maggio and Segal, 2009a, b), which could explain the disruption of the cue fear conditioning in the present study.

Using more physiological conditions, our last experiment shows that post-training (restraint) stress or systemic injection of CORT mimicked the effects of local CORT injections on fear memories. More specifically, we had previously shown that in a *predicting-context* situation, post-training stress or systemic CORT injection reproduced the effects of intra-DH CORT injections, i.e., enhancing adaptive contextual fear memory after a low stress condition, but producing a false tone fear memory after a high stress condition (Kaouane et al., 2012; see Figure 4A). In contrast, the present study shows that in a *predicting-cue* situation, the same manipulations reproduced the effects of intra-VH CORT injections, i.e., promoting an adaptive tone fear memory after a low stress condition while promoting a maladaptive contextual fear memory after a high stress condition. Under the low stress condition, the increase in tone fear memory is in accordance with previous studies showing that post-training glucocorticoid injections increase cued fear memory in low or mild stress situations (Hui et al., 2004; Marchand et al., 2007). Under the high stress condition, the observed CORT-induced maladaptive contextual fear memory and deficit in cue fear conditioning strongly suggest that activation of GR, in the VH specifically, constitutes a key molecular device for such fear memory disturbances. Now, how can we explain that the same biological manipulation (GR activation) can have drastically different, and even opposite, effects on fear memory as a function of the learning procedure used? Even if GRs respond similarly to systemic CORT/DEX injection in the DH and VH, the deleterious impact of this systemic injection on fear behavior is supposed to differ as a function of the specific recruitment of the DH and VH in the training procedure considered. Because the DH and VH are known to be differentially involved in contextual and tone fear conditioning, the present findings suggest that the opposite effect of systemic CORT/DEX injection on fear behavior may result from an imbalance between the recruitment of the DH and that one of the VH as a function of the training procedure used.

In conclusion, our study shows that glucocorticoids alter fear memories in an opposite way as a function of the hippocampal sector where GR are activated, providing evidence for a functional dissociation between the DH and the VH. These findings indicate that glucocorticoids, under high stressful situation, can produce false fear memories on the basis of the erroneous selection of the most salient, but irrelevant, simple cue or the background context as predictor of an aversive event. Classically, “false memory” applies to memories formed without the actual experience of the items that constitute the object of these memories. Here, “false memories” refer to totally erroneous memory representation of the stressful situation as regards to the objective training situation. Indeed, animals wrongly attribute an aversive predictive value to the salient but not predictive tone instead of the (foreground) context in the unpairing situation, and to the (overshadowed) context instead of the predictive tone in the pairing situation. These erroneous representations, based on distorted meanings of both the salient tone and the context, are clearly akin to “false memories.” These opposing false fear memories might be related to the development of different stress-related disorders. On the one hand, in human, specific alterations in the posterior hippocampus (DH in rodents) have been linked to PTSD-related cue-based hypermnesia and contextual amnesia (Brewin, 1996; Brewin and Holmes, 2003; American Psychiatric Association, 2013). We precisely reproduced this paradoxical memory alteration with intra-DH CORT (Kaouane et al., 2012) or DEX infusions in mice. On the other hand, the anterior hippocampus in human (Satpute et al., 2012) and the VH in rodents (Degroot and Treit, 2004) have been involved in anxiety-related behaviors including intense and irrational fear reactions to particular stressful situation (American Psychiatric Association, 2013). Such abnormally high fear behaviors to (background) contextual elements under stress were precisely those observed after intra-VH CORT or DEX infusions in the present study. Furthermore, highlighting different roles for the DH and the VH in *adaptive* and *maladaptive* (anxiety-like) contextual fear, respectively, our findings are congruent with an optogenetic study reporting similar functional dissociation (i.e., contextual fear vs. anxiety-like behaviors) along the dorso-ventral axis of the dentate gyrus (Kheirbek et al., 2013). Altogether, our findings strongly suggest the different effects of glucocorticoids induced by their action along the dorso-ventral axis of the hippocampus might explain the constellation of memory alterations observed after stressful situations, thereby contributing to a better understanding of the pathophysiology of stress-related disorders.

## Data Availability Statement

The raw data supporting the conclusions of this article will be made available by the authors, without undue reservation.

## Ethics Statement

The animal study was reviewed and approved by Bordeaux ethic committee; European Communities Council Directive (86/609/EEC).

## Author Contributions

NK, E-GD, and AD conceived, designed, performed the experiments, and analyzed the data. NK, AM, MS, and AD wrote the article.

## Conflict of Interest

The authors declare that the research was conducted in the absence of any commercial or financial relationships that could be construed as a potential conflict of interest.
