# Supplementary material for: False Opposing Fear Memories Are Produced as a Function of the Hippocampal Sector Where Glucocorticoid Receptors Are Activated
Source: Front Behav Neurosci. 2020 Aug 26;14:144. doi: 10.3389/fnbeh.2020.00144 (PMC7479235; doi:10.3389/fnbeh.2020.00144)
Supplement: Supplementary file 1 [file Data_Sheet_1.PDF]

Supplementary Material

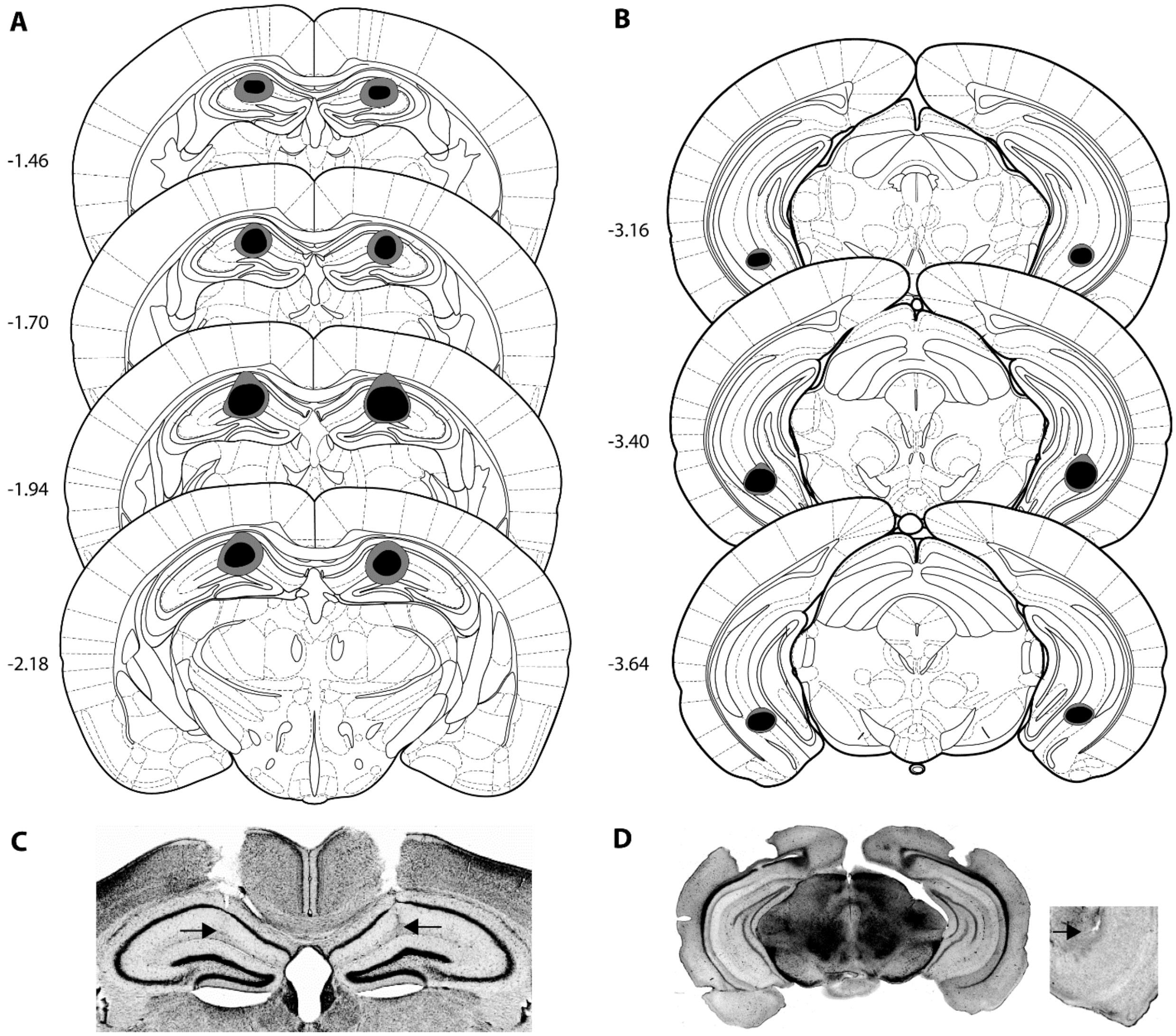

Supplementary Figure 1. Histological controls. Schematic representation of the area where the cannulas tip location were observed (black area) and largest sphere of drug diffusion (gray area) of a 0.3-µl or 0.1-µl solution of India ink into the DH **(A)** and VH **(B)**, respectively. Adapted from the Mouse Brain Atlas (Franklin and Paxinos, 2007). Representative photomicrographs depicting the guide cannulae placement and infusion sites (indicated by arrows) into the DH **(C)** and VH **(D)**.

Franklin, K.B.J., and Paxinos, G. (2007). The Mouse Brain in Stereotaxic Coordinates (Third Edition: Academic Press).

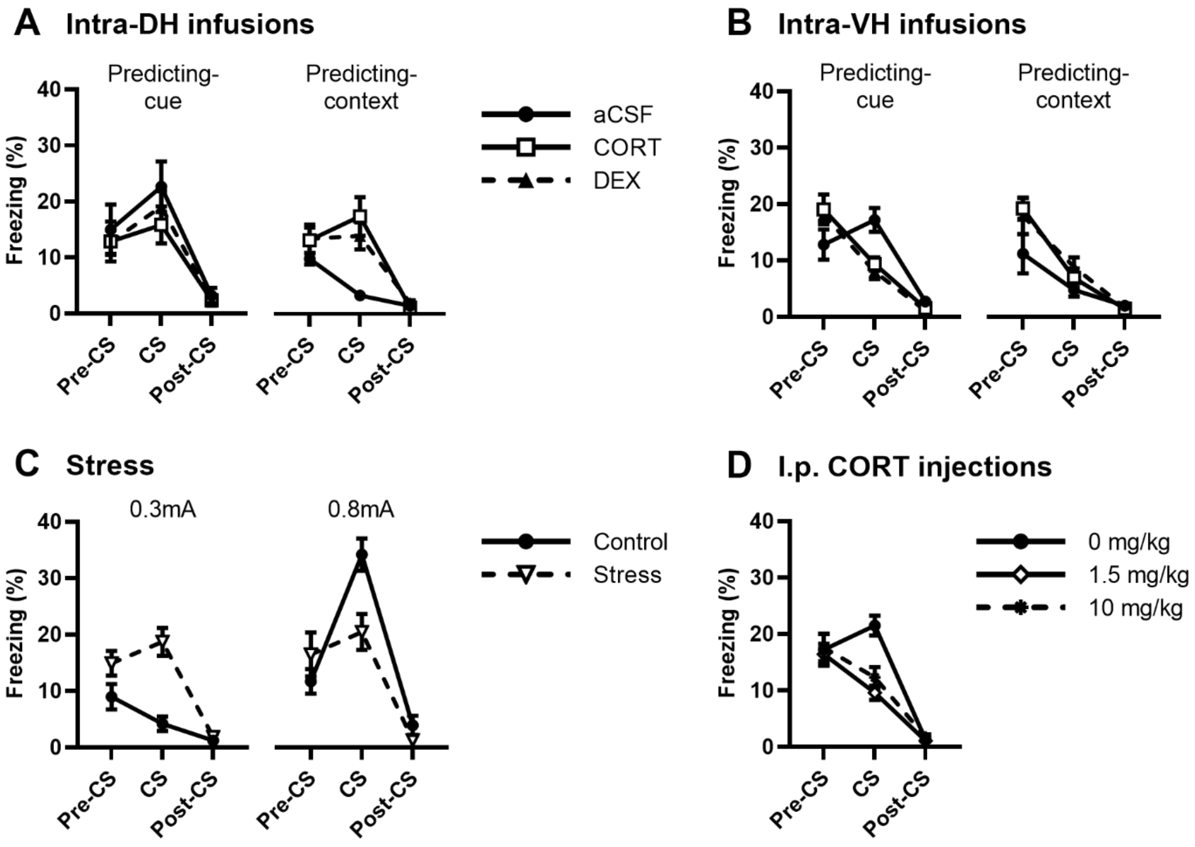

Supplementary Figure 2. Full results of the tone CS test. Mean percentage freezing (±SEM) on successive 2-min periods before (pre-CS), during (CS) and after (post-CS) tone CS presentation in animals that received intra-DH **(A)** or intra-VH **(B)** infusions, or submitted to post-training stress **(C)** or i.p. injections **(D)**.
